# Supplementary material for: Structural mechanism of bacteriophage lambda tail’s interaction with the bacterial receptor
Source: Nat Commun. 2024 May 17;15:4185. doi: 10.1038/s41467-024-48686-3 (PMC11101478; doi:10.1038/s41467-024-48686-3)
Supplement: Supplementary file 1 — Supplementary Information [file 41467_2024_48686_MOESM1_ESM.pdf]

Supplementary information for

Structural mechanism of bacteriophage lambda tail's interaction with the bacterial receptor

This PDF file includes:

Supplementary Figures S1 to S6
Supplementary Tables S1

Supplementary Figures

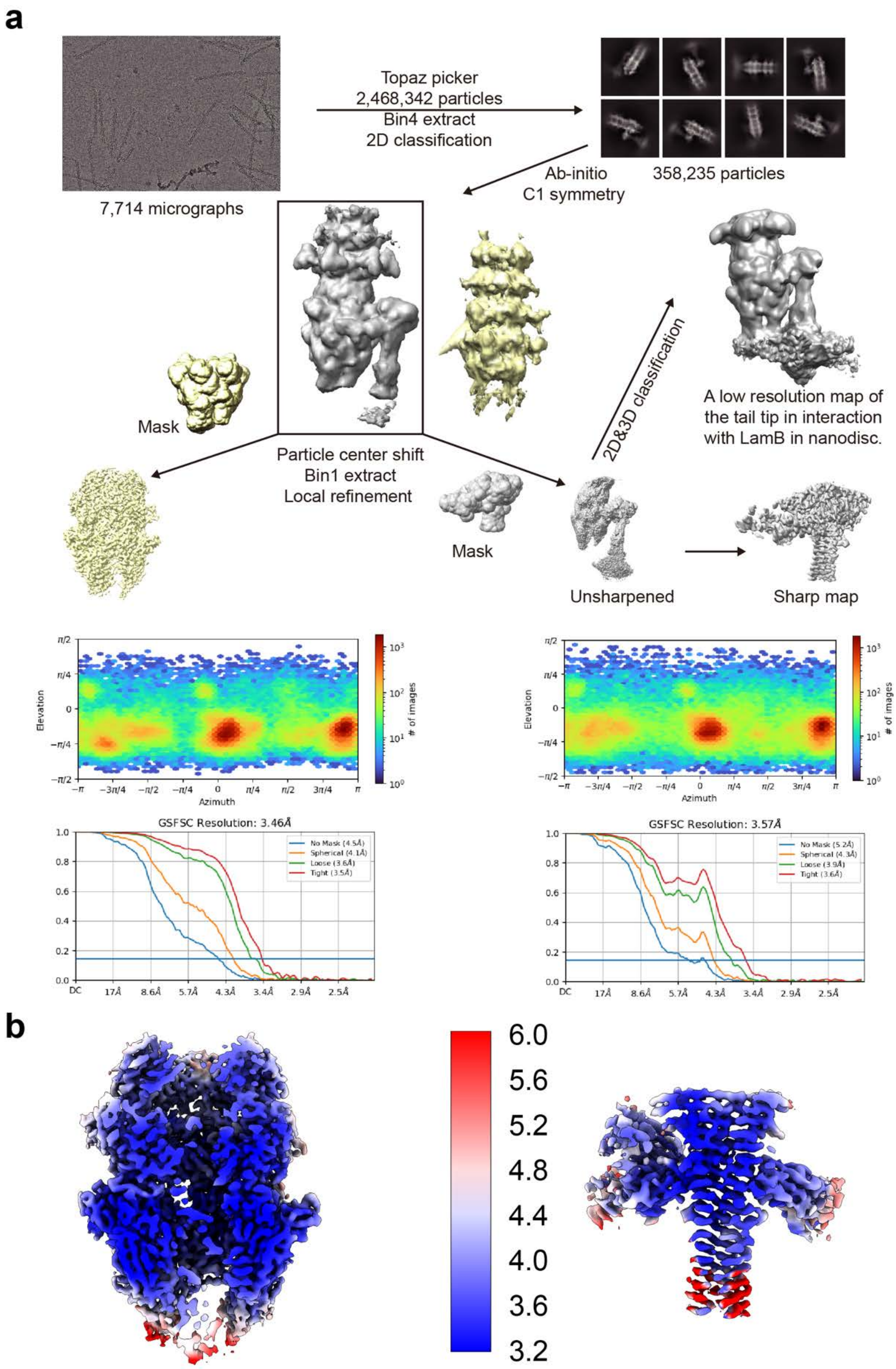

Figure S1. Flowchart of the EM processing pipeline of LamB nanodisc-incubated tail complex.

a Workflow diagram for the *S. sonnei* LamB nanodisc-incubated tail complex. An unsharpened map reveals subtle outlines of the RBD and LamB, and features remain identifiable despite the absence of sharpening. After further classification of the unsharpened map, A low-resolution map of the tail tip interacting with LamB in a nanodisc was obtained. Bottoms are the particle angular distribution in the final reconstruction and the gold-standard Fourier shell correlation (FSC)[1] curve of the 3D reconstructions.

b Local resolution maps of different parts of LamB nanodisc-incubated tail complex.

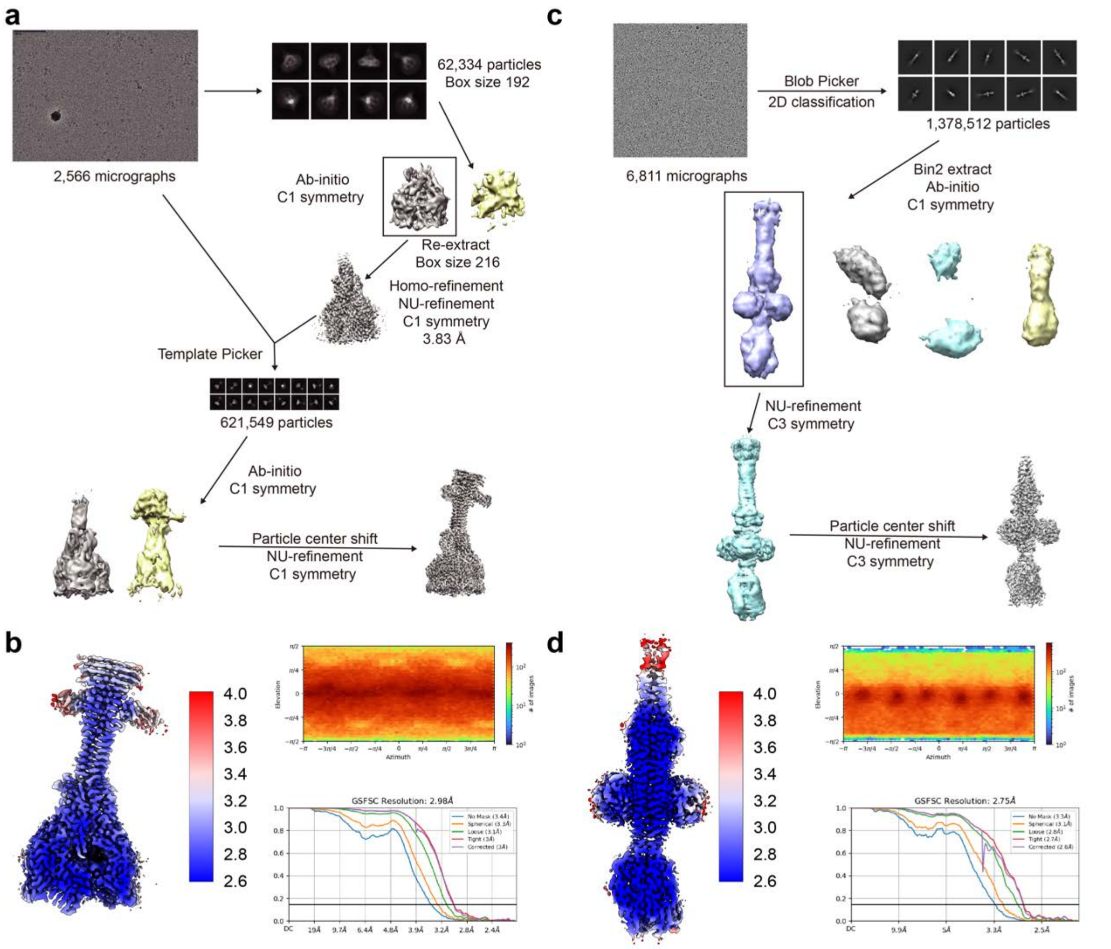

Figure S2. Flowchart of the EM processing pipeline of gpJ713 and ssLamB complex and gpJ713.

a Workflow diagram for the gpJ713 and ssLamB complex.

b The local resolution map, particle angular distribution in the final reconstruction and the gold-standard Fourier shell correlation (FSC)[1] curve of the 3D reconstructions in (a).

c EM processing workflow diagram for the gpJ713.

d The local resolution map, particle angular distribution in the final reconstruction and the gold-standard Fourier shell correlation (FSC)[1] curve

of the 3D reconstructions in (c). The closed gpJ713 structure inadvertently co-purified with the peptidylprolyl isomerase protein during purification.

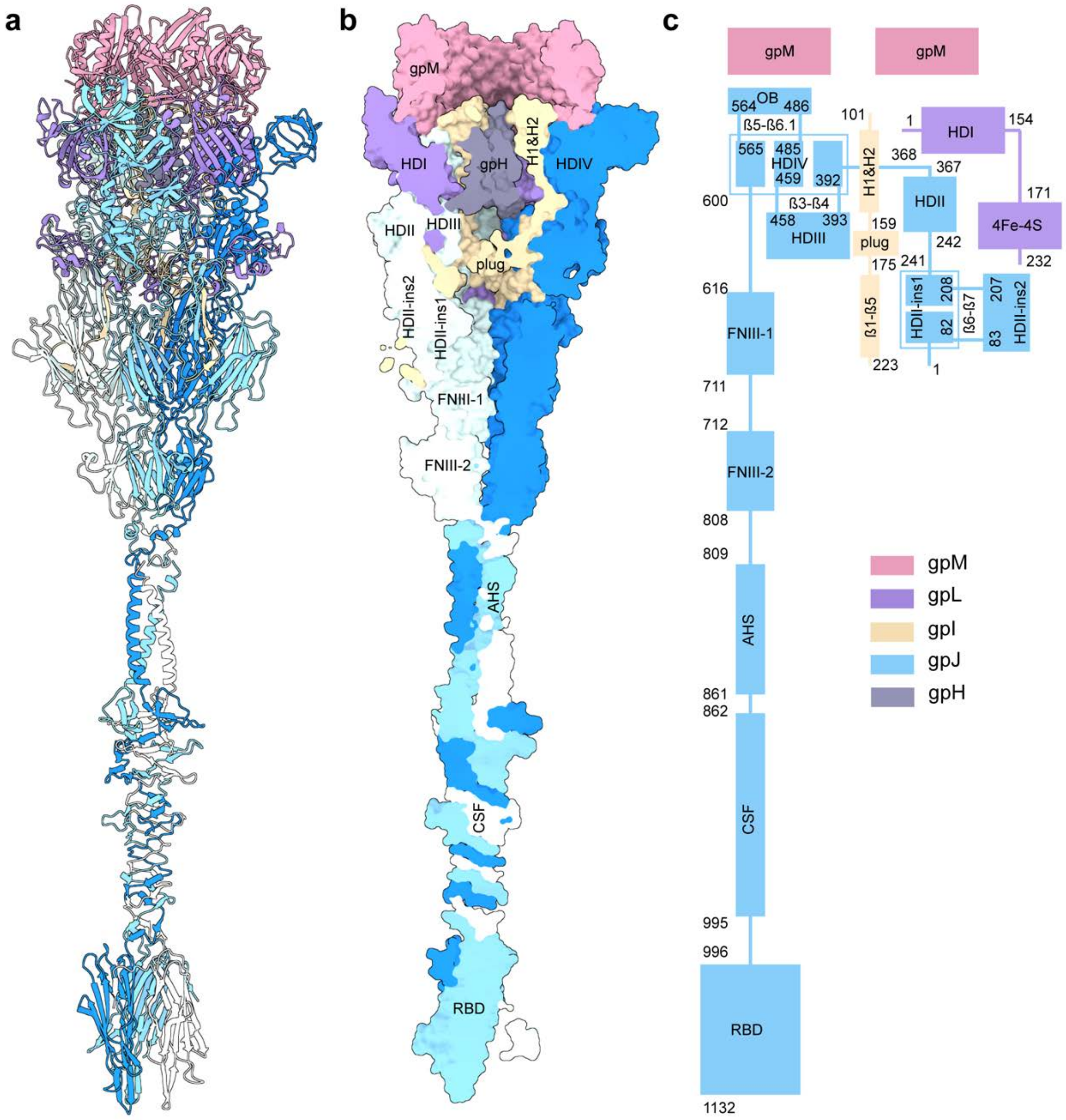

Figure S3. Topology and structure of closed lambda phage tail tip and fiber.

a Overall model of closed lambda phage tail tip and fiber.

b Central slice sideview of closed lambda phage tail tip and fiber.

c Topology diagram of closed lambda phage tail tip and fiber..

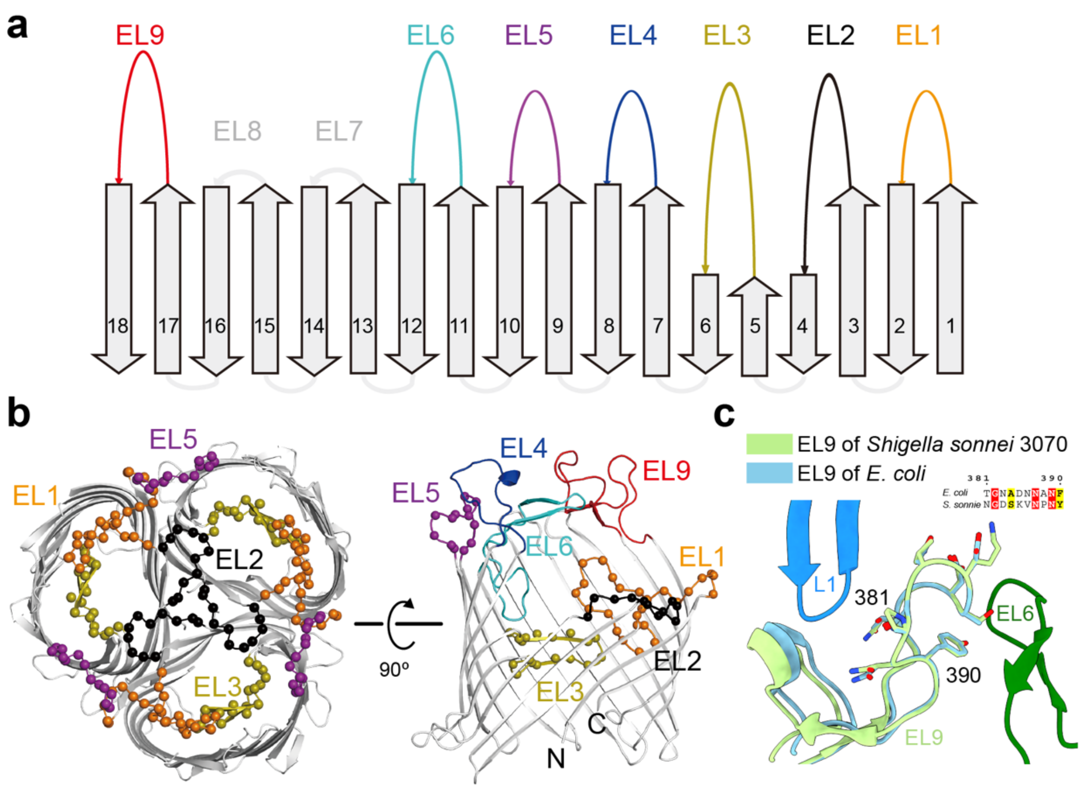

Figure S4. **Topology and structure of LamB.**

a Topology diagram of LamB. The extracellular loops are designated with numbers EL1-EL9.

b Top view of trimeric LamB. The oligomerization of the LamB trimer is facilitated by the involvement of specific loops, namely EL1, EL2, EL3, and EL5.

c A comparison of the EL9 structures in ecLamB and ssLamB reveals consistent overall structures, despite seven differences within residues 381-390.

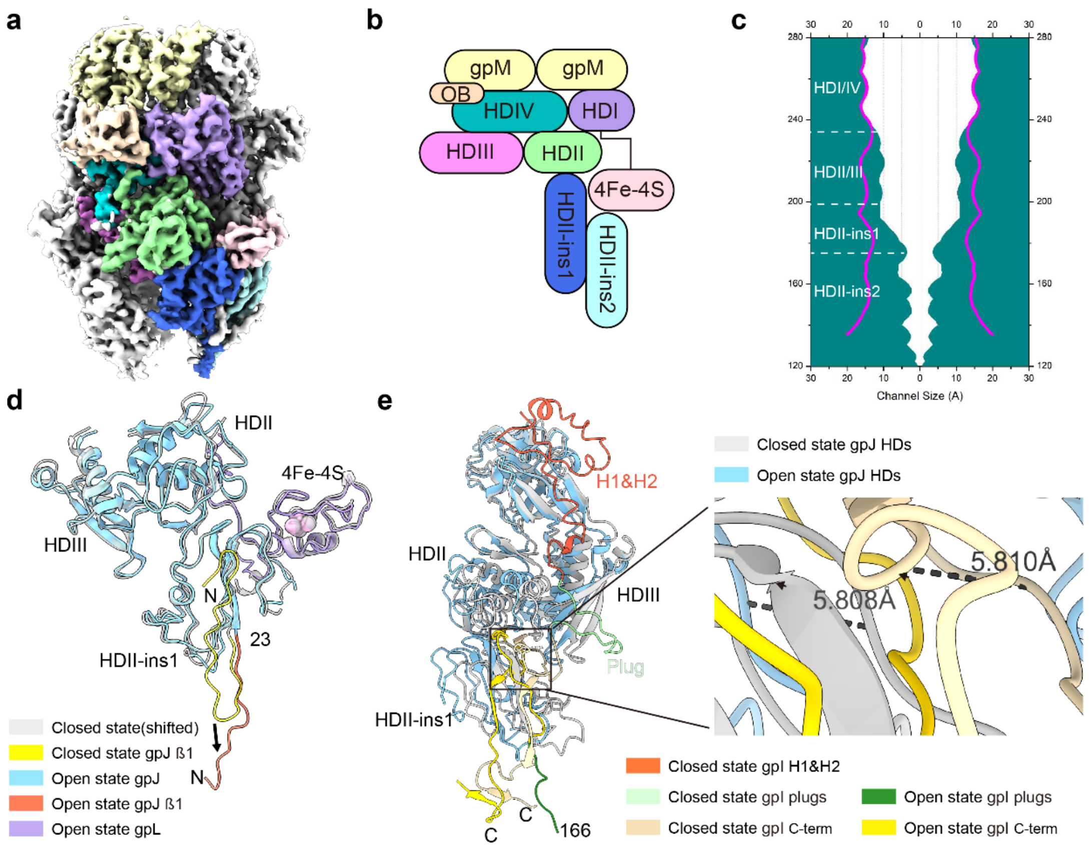

Figure S5. **Structural changes in the HDs of gpJ and gpL**

a Cryo-EM map of the tail tube in the open state. Domains of gpJ, gpL, and gpM are differentiated by distinct colors.

b Diagram depicting the domain organization of the tail tube, with coloring consistent with (a).

c Representation of the channel radii along the potential transport path. The boundary of the white region denotes the tube's internal diameter in the close state, while the pink lines illustrate the internal diameter following the movement of the HDs, computed using HOLE software[2].

d Alignment of the structures of the close (gray cartoon) and open states. The HDII, HDIII, HDII-ins1, and 4Fe-4S align well in both states, indicating they have undergone a rigid body shift. In the open state, the N-

terminal segment of the HDII-ins1 domain in the gpJ protein undergoes an approximately 180° flip.

e Correlation between the movement of gpJ's HD domain and the transit of gpI through it. In the left image, segments of gpJ's HD domains are portrayed in gray and blue for the close and open states respectively. The HDII, HDIII, and HDII-ins1 (excluding the N-terminal part in (d)) undergo a rigid body rotation as a unit. In the zoomed-in right image, it is underscored that the gpI segment passing through gpJ's aperture displaces in perfect alignment with the movement of gpJ's HDs.

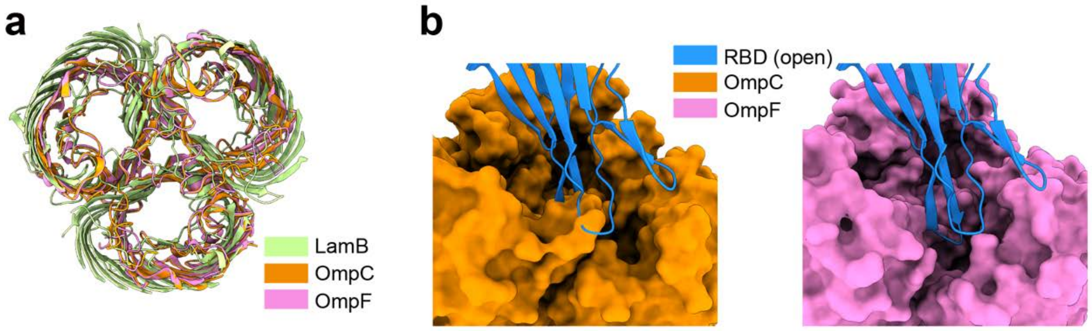

Figure S6. LamB, OmpC, OmpF interaction ability with gpJ RBD

a An overlaid topview slice of OmpC, OmpF, and LamB. The β-barrel regions of OmpC and OmpF are similar in size, yet both are smaller than LamB's (PDB ID: 8XCJ).

b OmpC (PDB ID: 2J1N)[3] and OmpF (PDB ID: 2ZFG)[4] are colored in orange and pink respectively. The blue loop area of gpJ clashes with the surface of OmpC, but not with OmpF.

Supplementary Tables

Table S1. Cryo-EM data collection, refinement and validation statistics

	**Tail-LamB (tail tip)**	**Tail-LamB (tail fiber)**	**gpJ713-LamB**	**gpJ713**
**Data collection and processing**				
Magnification	81,000		81,000	75,000
Voltage (kV)	300		300	300
Electron exposure ($e^-/Å^2$)	50		50	50
Defocus range (μm)	-1.5 – -2.5		-1.5 – -2.5	-1.5 – -2.5
Pixel size (Å)	1.0742		1.0742	1.036
Micrographs (no.)	7,714		2,566	6,811
Symmetry imposed	C1	C1	C1	C3
Final particle images (no.)	358,235		417,370	369,942
Map resolution (Å) 0.143 FSC threshold	3.46	3.57	2.98	2.75
Map sharpening B factor ($Å^2$)	-118.9	-129.2	-118.3	-108.1
**Refinement**				

CC (model vs. data)	0.7918	0.6992	0.8872	0.8149
Model composition				
Chain count	15	3	6	6
Non-hydrogen atoms	25,888	8,822	19,809	10,665
Protein residues	3,312	1,143	2,526	1,395
Ligands	24	0	0	0
B factors (Å^2)				
Proteins	136.161	170.557	41.654	32.774
Ligands	109.367	---	---	---
R.m.s. deviations				
Bond lengths (Å)	0.0038	0.004	0.0031	0.003
Bond angles (°)	0.73	0.73	0.60	0.676
Validation				
MolProbity score	2.87	2.92	2.31	2.39
Clashscore	13.59	19.39	8.92	10.54
Poor rotamers (%)	6.7	7.62	5.93	2.49
Ramachandran plot				
Favoured (%)	87.81	92.66	96.3	90.02
Allowed (%)	11.43	6.98	3.34	9.98

Disallowed (%)	0.76	0.35	0.36	0.0
PDB code	8XCG	8XCI	8XCJ	8XCK
EMDB code	EMD-38242	EMD-38244	EMD-38245	EMD-38246

References

1 Rosenthal, P. B. & Henderson, R. Optimal determination of particle orientation, absolute hand, and contrast loss in single-particle electron cryomicroscopy. *J Mol Biol* **333**, 721-745 (2003). https://doi.org:10.1016/j.jmb.2003.07.013

2 Smart, O. S., Neduvelil, J. G., Wang, X., Wallace, B. A. & Sansom, M. S. HOLE: a program for the analysis of the pore dimensions of ion channel structural models. *J Mol Graph* **14**, 354-360, 376 (1996). https://doi.org:10.1016/s0263-7855(97)00009-x

3 Baslé, A., Rummel, G., Storici, P., Rosenbusch, J. P. & Schirmer, T. Crystal structure of osmoporin OmpC from E. coli at 2.0 A. *J Mol Biol* **362**, 933-942 (2006). https://doi.org:10.1016/j.jmb.2006.08.002

4 Yamashita, E., Zhalnina, M. V., Zakharov, S. D., Sharma, O. & Cramer, W. A. Crystal structures of the OmpF porin: function in a colicin translocon. *Embo j* **27**, 2171-2180 (2008). https://doi.org:10.1038/emboj.2008.137
