# Supplementary material for: Structural mechanism of bacteriophage lambda tail’s interaction with the bacterial receptor
Source: Nat Commun. 2024 May 17;15:4185. doi: 10.1038/s41467-024-48686-3 (PMC11101478; doi:10.1038/s41467-024-48686-3)
Supplement: Supplementary file 3 — Description of Additional Supplementary Files [file 41467_2024_48686_MOESM3_ESM.pdf]

Description of Additional Supplementary Files

Supplementary Movie 1. Conformational changes occurring in the AHS, CSF, and RBD regions as the bacteriophage lambda tail transitions from a closed to an open state. The initial position corresponds to the closed state (PDB: 8XCK), while the final position represents the open state (PDB: 8XCJ).
